# Protein-linked glycans in periodontal bacteria: prevalence and role at the immune interface

**DOI:** 10.3389/fmicb.2013.00310

**Published:** 2013-10-17

**Authors:** Rajendra P. Settem, Kiyonobu Honma, Graham P. Stafford, Ashu Sharma

**Affiliations:** ^1^Department of Oral Biology, School of Dental Medicine, University at Buffalo, State University of New YorkBuffalo, NY, USA; ^2^Oral and Maxillofacial Pathology, School of Clinical Dentistry, Claremont Crescent, University of SheffieldSheffield, UK

**Keywords:** protein glycosylation, periodontal bacteria, immune response

## Abstract

Protein modification with complex glycans is increasingly being recognized in many pathogenic and non-pathogenic bacteria, and is now thought to be central to the successful life-style of those species in their respective hosts. This review aims to convey current knowledge on the extent of protein glycosylation in periodontal pathogenic bacteria and its role in the modulation of the host immune responses. The available data show that surface glycans of periodontal bacteria orchestrate dendritic cell cytokine responses to drive T cell immunity in ways that facilitate bacterial persistence in the host and induce periodontal inflammation. In addition, surface glycans may help certain periodontal bacteria protect against serum complement attack or help them escape immune detection through glycomimicry. In this review we will focus mainly on the generalized surface-layer protein glycosylation system of the periodontal pathogen *Tannerella forsythia* in shaping innate and adaptive host immunity in the context of periodontal disease. In addition, we will also review the current state of knowledge of surface protein glycosylation and its potential for immune modulation in other periodontal pathogens.

## INTRODUCTION

Until relatively recently, protein glycosylation was believed to be restricted only to eukaryotic proteins. However, with the availability of genome-scale information and sophisticated mass spectrometry as well as nuclear magnetic resonance techniques primarily from the past decade it has come to light that protein glycosylation occurs quite frequently in bacteria as well (Nothaft and Szymanski, 2010). More and more bacterial proteins of Gram-positive and Gram-negative origin are frequently being identified with *N*- and *O*-linked glycan modifications (Ashline et al., 2005; Brisson et al., 2010; reviewed in Iwashkiw et al., 2012, 2013). Moreover, it is becoming apparent that protein glycosylation is also prevalent amongst Archaea (Eichler, 2013; Kandiba and Eichler, 2013). Dedicated computational algorithms have also been developed to predict glycosylated residues in prokaryotic proteins (Chauhan et al., 2012), and a web program, GlycoPP at , using such algorithms is currently available for predicting *N*- and *O*-glycosylation sites in prokaryotic protein sequences with an accuracy of 70–80%. In most cases bacterial glycans are linked directly to asparagine or serine/threonine residues within specific peptide motifs for *N*- and *O*-glycosylation, respectively. Proteins such as flagellins, pilins, and surface (S)-layer proteins have been shown to be glycosylated quite extensively in bacteria by dedicated glycosylation systems (Nothaft and Szymanski, 2010). Recent studies have alluded to the role of protein glycosylation in immune modulation in pathogenic as well as commensal bacteria, such as in *Neisseria* species (Vik et al., 2009), *Campylobacter jejuni* (Szymanski and Wren, 2005), and *Bacteroides fragilis* (Fletcher et al., 2009), and in attachment and infective ability of *Chlamydia trachomatis* (Kuo et al., 1996) and *C. jejuni* species (Szymanski et al., 2002; Karlyshev et al., 2004). In addition, variations in flagellar protein-linked glycans in *C. jejuni* have been shown to cause phenotypic phase-variations and antigenic diversity, important for the pathogen’s virulence (Guerry et al., 2006; Young et al., 2007; Hitchen et al., 2010).

**FIGURE 1:**
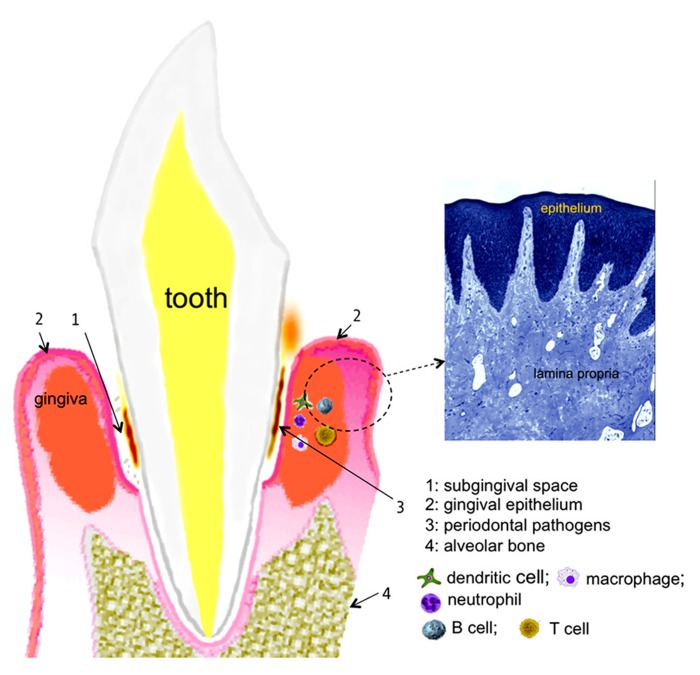
**chematic representation of the periodontal tissues supporting the tooth.** A histological section of the gingival tissue stained with toluidine blue is shown on the right (courtesy, Dr. Moon-Il Cho, University at Buffalo).

With respect to the human oral cavity, the niche is colonized with nearly 700 bacterial species with different taxa and phylotypes (Dewhirst et al., 2010). Of these diverse bacteria, a select group of Gram-negative anaerobes residing in the subgingival crevices (spaces between the teeth and gums; **Figure 1**) as biofilms induce one of the most common forms of the inflammatory disease in humans, known as periodontitis. This disease is characterized by the progressive destruction of the tooth supporting apparatus and often leads to tooth loss in adults. In addition, chronic infections with periodontal pathogens have been linked to the risk for systemic health conditions such as cardiovascular, diabetic, respiratory, and arthritic diseases (Beck et al., 2008; Genco, 2009; Gomes-Filho et al., 2009; Genco and Van Dyke, 2010; Scher et al., 2012). A group of bacteria known as the “red complex” comprising *Porphyromonas gingivalis*, *Treponema denticola*, and *Tannerella forsythia* has been strongly implicated in the initiation and progression of periodontitis (Socransky et al., 1998). We discuss below recent data mainly from studies in *T. forsythia* and *P. gingivalis* that suggest these bacteria utilize protein glycosylation for undermining the host immunity to persist in the host and cause periodontal destruction.

## PROTEIN-LINKED GLYCANS OF PERIODONTAL BACTERIA AS IMMUNOMODULATORS

**FIGURE 2:**
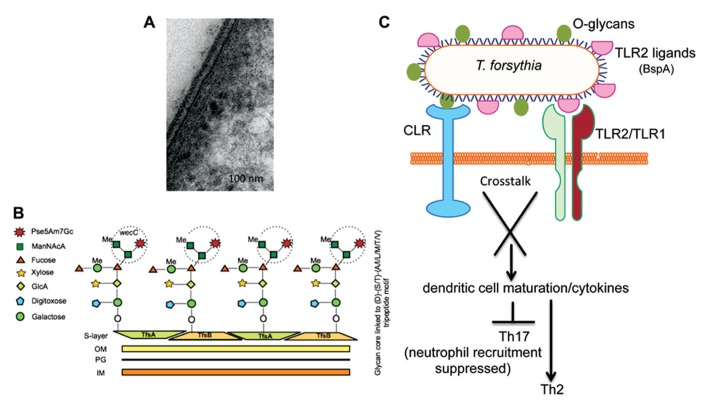
** (A)** Transmission electron microscopy (TEM) micrographs of *T. forsythia* ultrathin sections. **(B)** Schematic diagram of the *O*-glycan core structure linked to a peptide motif in *Tannerella* surface-layer proteins. A terminal trisaccharide motif synthesized by the *wecC* operon is circled. **(C)** Signaling crosstalk between a C-type lectin-like receptor (CLR) and TLR2 activated by *O*-glycans and TLR2 ligands (e.g., BspA), respectively, may orchestrate T helper cell differentiation.

The periodontal pathogen *T. forsythia* possesses a S-layer, a tooth-like serrated layer covering the outer-membrane (Sabet et al., 2003; **Figure 2A**). S-layers are comprised of water insoluble proteins intrinsically capable of self-assembling into crystalline lattice and are believed to provide protection against the deleterious effects of the environment as well host immune components (Sara and Sleytr, 2000). S-layer proteins in Gram-positive bacteria may be glycosylated by the addition of *O*-linked long-chain glycan repeats in a process similar to lipopolysaccharide (LPS) biosynthesis (Messner et al., 2008). The *T. forsythia* S-layer, however, is unique in that it is the only Gram-negative S-layer known to date which is glycosylated (Lee et al., 2006). It is comprised of two high molecular weight glycoproteins, TfsA (200 kDa) and TfsB (210 kDa) which are clearly prominent in the protein profiles of this organism and make up a large proportion of the total bacterial protein.

Our previous work and that of the Schaffer group (using our *wecC* mutant) showed that a putative exopolysaccharide synthesis operon *wecC-neuC-gtf* is involved in the *O*-glycosylation of *Tannerella* S-layer and other surface proteins (Honma et al., 2007; Posch et al., 2011). Specifically, the *wecC* gene product (UDP-N-acetylmannosaminuronic acid dehydrogenase homolog) was shown to be involved in the addition of a three-sugar-branch motif comprising two sub-terminal mannosuronic acid (ManNAcA) and a terminal modified pseudaminic acid residues (Pse5Am7Gc, 5-acetimidol-7-*N*-glycolyl-pseudaminic acid) on an *O*-linked oligosaccharide core attached to the surface glycoproteins (Posch et al., 2011; **Figure 2B**). The glycan core is attached to the S-layer protein (and several other *Tannerella* surface proteins) at several sites in each protein via *O*-linkages at Ser, Thr, or Tyr residues within the three amino acid motif D(S,T)(A,L,V,I,M,T) (Posch et al., 2011). This mode of glycosylation at specific three amino acid motifs appears to be well conserved across a range of Bacteroidetes species. This is evidenced by elegant transcomplementation experiments in which the *T. forsythia* S-layer protein was produced and glycosylated at this motif with the *B. fragilis* specific glycan, and vice versa, i.e., a *B. fragilis* protein was glycosylated in *T. forsythia* with the *Tannerella* glycan (Posch et al., 2013). In fact this has now been shown to be the case in a range of *Bacteroides*, *Prevotella*, and *Porphyromonas* species with bioinformatic (Fletcher et al., 2011) as well as experimental approaches using an antibody against the core component of the *Bacteroides* group *O*-glycan (Coyne et al., 2013). This conservation of a general *O*-glycosylation system in Bacteroidetes is indicative of its potentially essential function in divergent Bacteroidetes species living in wide-ranging niches.

Previous studies have shown that the *Tannerella* S-layer is capable of mediating bacterial attachment to and invasion of epithelial cells (Lee et al., 2006). Furthermore, for the benefit of the bacterium, the S-layer helps to blocks the early release of inflammatory cytokines by immune cells. The role of S-layer glycosylation on the bacterium’s ability to directly modulate host immunity to impact its *in vivo* life-cycle has now come to light. A recent study from our laboratory showed that loss of the terminal trisaccharide branch motif on the S-layer as well as a number of other *O*-glycosylated surface proteins due to *wecC* deficiency renders the bacterium increasingly prone to recognition and processing by antigen-presenting cells (Settem et al., 2012). Specifically, the loss of this terminal tri-saccharide branch (**Figure 2B**) promotes phagocytosis of the bacterium by a subset of antigen presenting cells called dendritic cells (DCs) and concurrent induction of Th17-activating cytokines IL-6 and IL-23 (Settem et al., 2012). This modulation of DC responses has major consequences for pathogenesis since DCs reside as immune system sentinels within tissues that constantly encounter invading microbes. In tissues they recognize different pathogen-associated molecular patterns (PAMPs) and act to direct innate and adaptive immune responses locally via pathogen-recognition receptors (PRRs), a group of molecules which include Toll-like receptors (TLRs), nucleotide oligomerization domain receptors (NODs), and C-type lectin-like receptors (CLRs; Takeuchi and Akira, 2010). It is the latter of these, the CLRs, which are most pertinent to the recognition of bacterial *O*-glycans since their function is to act as carbohydrate binding receptors. Thus, surface *O*-linked glycans, the tri-saccharide motif in particular, impact DC-mediated processing of the bacterium, also impacting the activation of intracellular receptors and inflammasome processing for IL-23 induction. This manipulation of DC behavior via carbohydrate ligands is a growing theme in bacterial pathogen biology and several recent studies indicate that bacterial polysaccharides can block recognition and phagocytosis of microbes by DCs and macrophages (Meijerink et al., 2012). Moreover, microbe-associated glycans regulate DC maturation and cytokine secretion to affect polarization of naïve T cells into specific subtypes upon microbial infection, with signaling crosstalk between CLRs and TLRs thought to influence this development (Geijtenbeek and Gringhuis, 2009). In the case of *T. forsythia*, *O*-glycan-mediated signaling via a CLR might synergize with TLR2-dependent signaling induced by the bacterium’s major virulence factor and TLR2 ligand, the BspA protein (Onishi et al., 2008; Myneni et al., 2012; **Figure 2C**).

Indeed, in line with the above predictions, the terminal *O*-glycan motif attached to the surface proteins of *T. forsythia* does seem to be a key factor in influencing T cell polarization during *T. forsythia* infection. The evidence to support this comes from data generated in mouse models infected with wild-type bacteria (decorated with the *O*-glycan) which induce robust TLR2-dependent Th2-biased inflammatory responses, leading to alveolar bone resorption (Myneni et al., 2011). Strikingly, this TLR2-Th2 bias is perturbed in response to infection with a bacterial strain lacking the terminal tri-saccharide *O*-glycan (i.e., a *wecC* mutant, **Figure 2B**) and when mice are infected with the strain lacking this terminal three-sugar branch motif on the *O*-linked glycan core, T cells polarize into the Th17 subset (Settem et al., 2012). This leads to increased infiltration of neutrophils into the gingival tissues and therefore decreased persistence of bacteria in periodontal tissues, resulting in reduced alveolar bone loss compared to the mice infected with the wild-type strain (Settem et al., 2012). Taken together, these data suggest that the surface *O*-glycan motif of *T. forsythia* is involved in suppressing Th17 responses, which allow increased bacterial persistence, promoting Th2-dependent inflammatory alveolar bone destruction (**Figure 2C**).

The other “red complex” periodontal pathogen *P. gingivalis* possesses two types of cell-surface fimbriae, the major fimbria known as FimA and the minor fimbria known as Mfa1. Mfa1 is comprised of a 67-kDa protein with *N*- and *O*-linked fucose, mannose, *N*-acetylglucosamine, and *N*-acetylgalactosamine sugars (Zeituni et al., 2010). Mfa1 activates DC-SIGN (dendritic cell-specific intercellular adhesion molecule-3-grabbing non-integrin) lectin on DCs. On the other hand, the 43-kDa major fimbrial protein FimA is non-glycosylated and activates TLR2. Recent studies further suggest that a crosstalk between TLR2 and DC-SIGN signaling due to FimA and Mfa1 fimbria, respectively, modulates the expression of Th cell differentiation cytokines in DCs against *P. gingivalis*, and is thus thought to orchestrate specific T cell immunity (Zeituni et al., 2010).

Orchestration of Th bias through the mediation of glycan modifications is not confined to periodontal pathogens alone, but is widely observed in the gastrointestinal pathogens and commensals. In the case of *C. jejuni*, surface capsular polysaccharide modifications with methyl phosphoramidate have been shown to modulate Th17 immunity against the pathogen and thus impact bacterial persistence in the host (Maue et al., 2012). With respect to the ubiquitous commensal and opportunistic pathogen – *B. fragilis*, the expression of its polysaccharide antigen (PSA) helps to keep inflammatory T cell lineages (e.g., Th17) in check and simultaneously promote IL-10-producing FoxP3(+) Treg cells in the gut (Mazmanian et al., 2008; Ochoa-Reparaz et al., 2010; Surana and Kasper, 2012). This role of PSA in influencing T cell populations is so profound that PSA-deficient bacteria skew the balance so far toward Th17 cells that infected mice develop colitis. Similar types of zwitterionic polysaccharides (ZPS) with immunomodulatory roles have also been identified in bacteria such as *Streptococcus* pneumoniae and *Staphylococcus aureus* type-5 and -8 (Tzianabos et al., 2000; Gonzalez-Fernandez et al., 2008). A clear mechanistic understanding of how bacterial polysaccharide regulate T cell polarization is lacking in light of the fact that PSAs are generally not presented to T cells (T cell independent) via antigen presenting cells. Rather, it has been demonstrated that ZPS antigens can be presented to T cells by APCs in an major histocompatibility complex class II (MHC-II) restricted manner (Avci and Kasper, 2010). This picture is further complicated by evidence suggesting that many oligosaccharides linked to peptides can directly activate T cells (Avci et al., 2013). Whether or not *Tannerella* polysaccharides are recognized by MHC-II or directly activate T cells is currently unknown. Alternatively, modulation of DCs through activation of CLRs such as DC-SIGN by *Tannerella* glycans could plausibly regulate the secretion of T cell biasing cytokines. In support of this notion, DC-SIGN is known to exhibit crosstalk with TLRs and impact the maturation of DCs and differentially regulate the cytokine environment, promoting polarization of T cells into specific subsets (Geijtenbeek and Gringhuis, 2009; Geijtenbeek et al., 2009; van den Berg et al., 2012).

## OTHER GLYCAN-RELATED FACTORS OF PERIODONTAL BACTERIA

It is also worth noting that several glycoproteins with potential roles in bacterial pathogenicity, including the arginine- (RgpB and RgpA) and lysine-specific (Kgp) cysteine proteinases (collectively known as gingipains) and major outer-membrane protein *pgm6* (OmpA) have been reported in *P. gingivalis* (Kishi et al., 2012). Gingipain proteases are major virulence factors of *P. gingivalis* that paralyze host immune defenses by degrading serum complement, antibodies and chemokines, and T cell biasing cytokines (Guo et al., 2010). Although glycans linked to gingipains *per se* do not play any direct role in corrupting host defense molecules, post-translation polysaccharide modifications via a novel protein secretion system (PorSS; Paramonov et al., 2005; Glew et al., 2012) as well as a Vim (virulence modulating) dependent glycosylation system (Aruni et al., 2013) play an essential role in the biogenesis of these proteases in *P. gingivalis*. In addition, recent work of Comstock and colleagues on the general *O*-glycosylation system in Bacteroidales species suggests the presence of a shared *O*-glycosylation system in a range of periodontal pathogens including *P. gingivalis* and at least two *Prevotella* spp. (Coyne et al., 2013). With respect to another major periodontal pathogen, *Treponema denticola*, a recent study points to the role of sialic acid modified surface glycoproteins in resisting the serum complement mediated killing of the pathogen (Kurniyati et al., 2013). However, basic information regarding the glycosylation machinery in this important pathogen is currently lacking. It is also worth noting that many species of *Campylobacter* associated with periodontitis (Macuch and Tanner, 2000), namely, *C. rectus, C. showae*, and *C. concisus* appear to also possess possible flagella glycosylation islands based on genome predictions (unpublished).

Furthermore, most intriguing is the presence of myriad glycosidases in the periodontal pathogen *T. forsythia* (Sharma, 2010), which otherwise depend on host proteinaceous components for growth. These include β-hexosaminidases (Roy et al., 2012) and most prominently sialidases (Braham and Moncla, 1992; Ishikura et al., 2003; Honma et al., 2011; Roy et al., 2011; Kurniyati et al., 2013) which appears to play a role in colonization and pathogenesis. We speculate that sialidases of periodontal bacteria could likely be involved in immune regulation as well, as is the case with the pneumococcal sialidase which can potentially induce leukocytes to release proinflammatory cytokines through relieving the dampening effect of Siglec receptors (Chang et al., 2012). Siglecs are sialic acid binding lectins on immune cells. Their activation by cognate sialoconjugates causes recruitment of phosphatases to the ITIM domain (immunoreceptor tyrosine-based inhibitory motif), leading to the inhibition of kinase-dependent activation of inflammatory signaling cascade in the cell (Crocker et al., 2007). Thus, hydrolysis of sialic acid from Siglec ligands by bacterial sialidases can potentially mitigate the immunosuppressive activity of Siglecs. Alternatively, sialidases may provide sialic acid to periodontal bacteria for incorporation into their cell-surface components, affording them the ability to undermine immune system in different ways. In *P. gingivalis*, a recent study demonstrated that bacterial sialidases play important roles in capsule formation and resistance to complement attack (Li et al., 2012).

## GLYCOMIMICRY

Evading immune recognition is critical for pathogen survival in the host. Pathogens decorate their surfaces with molecular structures that can mimic host molecules to avoid detection by the immune system. For example, hyaluronic acid and α2–8-linked sialic acid polymers are abundant on capsule polysaccharides of group-A streptococci (GAS) and group-B streptococci (GBS). These structures resemble glycosaminoglycan and glycosylated proteins present on host connective, epithelial and neural tissues respectively. The GBS-expressed polysaccharide mimics a unique glycan structure found in human glycoproteins recognized by Siglecs, coopting of which by bacterial polysaccharide can lead to dampening of the immune responses including the impairment of neutrophil attack. Likewise, polysaccharides of *Neisseria meningitidis* and *Escherichia coli* K1 antigens are structurally similar to those of human fetal neuronal cells (Finne et al., 1987; Robbins et al., 2011). It was also demonstrated that this microbial glycomimicry can mislead the host immune system. For instance, carbohydrate mimicry between *C. jejuni* lipooligosaccharide and gangliosides has been implicated in the onset of paralytic disorder Guillain–Barré syndrome. In this setting, infection by *C. jejuni* in humans is believed to induce cross-reactive serum antibodies which bind to and damage peripheral nerves resulting in autoimmune disorders (Yuki et al., 2004). Interestingly, *T. forsythia* terminal sugar pseudaminic acid linked to an *O*-glycan core at least partially mimics sialic acid structures. Therefore, it is tempting to speculate that by abundantly decorating the surface with modified pseudaminic acid residues the bacterium could avoid immune surveillance and enhance its survival.

## CONCLUSION

This review highlights the range and potential for immunomodulation by surface protein glycans in several periodontal bacteria. We also highlight the action of an exemplar glycan, namely that on the *Tannerella* S-layer that influences immune responses and promotes survival and persistence in the body while promoting bone loss orally. We hypothesize that bacterial glycans might have a prominent role in influencing local and systemic immunity to affect the persistence and pathogenicity of pathogenic oral communities in periodontal disease. Therefore, the future targeting of such interactions may provide a novel approach for modulating periodontitis.

## Conflict of Interest Statement

The authors declare that the research was conducted in the absence of any commercial or financial relationships that could be construed as a potential conflict of interest.
